# Intraoperative portable ultrasonography localization of clinically impalpable soft-tissue tumors

**DOI:** 10.1186/1477-7819-10-243

**Published:** 2012-11-13

**Authors:** Jagajeevan Jagadeesan, Jonathan A Davies, Anna Raurell, Robert U Ashford

**Affiliations:** 1 East Midlands Sarcoma Service, Nottingham City Hospital, Hucknall Road, Nottingham, NG5 1PB, UK; 2 East Midlands Sarcoma Service, Department of Orthopaedics, Leicester Royal Infirmary, Infirmary Square, Leicester, LE1 5WW, UK; 3 Division of Orthopaedic and Accident Surgery, University of Nottingham, Queens Medical Centre, Nottingham, NG7 2UH, UK

**Keywords:** Soft-tissue sarcomas, Intraoperative localization, Portable ultrasonography, Sonosite®

## Abstract

**Background:**

Most soft-tissue tumors are clinically palpable; however, some can be impalpable to clinical examination making it difficult to plan surgical management.

**Methods:**

We present a simple method of perioperative tumor localization using a portable ultrasonography machine.

**Results:**

We used the technique for seven cases, on each occasion identifying the tumor and facilitating the optimal surgical approach.

**Conclusion:**

The technique is reproducible and readily available, and we recommend its use.

## Background

Most soft-tissue tumors are obvious on clinical examination, but some (typically small and deep tumors) can be impalpable, making it difficult to plan surgical resection. A vital step in obtaining local control of soft-tissue sarcomas is the proper placement of the initial biopsy site to obtain tissue diagnosis, followed by an appropriately planned incision to enable the biopsy tract to be excised *en bloc* with the whole surgical resection specimen, to eradicate the possibility of tumor seeding along the biopsy track. Poorly placed incisions and biopsy complications can considerably affect the ability to achieve local clearance [1], and can result in amputation rather than limb salvage [2]. This is a particular problem with impalpable limb tumors, which often require a scan on the day of the surgery by an experienced musculoskeletal radiologist. This involves planning, and potentially results in the cancellation of the procedure if not organized in advance. The alternative is to make the surgical approach guided by MRI scanning, which typically results in a more extensile approach, which is large relative to the size of the tumor. In breast surgery, impalpable tumors are commonly excised using fine-wire localization.

Portable ultrasonography is readily available in most operating theatres across the UK. There is clear evidence and guidance on the use of ultrasonography to aid practitioners when performing nerve blocks and inserting central venous lines [3,4], and this has helped embed ultrasonography into anesthetic practice. Gaining competence in using ultrasonography is an essential part of anesthetic training in the UK, and it is guided by a joint working party of the Association of Anaesthetists of Great Britain and Ireland, the Royal College of Anaesthetists, and the Intensive Care Society [5]. Most anesthetists are therefore familiar and competent in using portable ultrasonography.

Because of its ease of availability, portable ultrasonography has been widely used in many specialties for bedside and intraoperative evaluation. It has been used intraoperatively for the successful placement of stents in pyeloplasty [6], in many emergency departments for identification of soft-tissue foreign bodies [7] and in the intraoperative evaluation of atheromatous disease in the aorta to prevent neurological complications in cardiac surgery [8].

We present a simple method of soft-tissue tumor localization using a portable ultrasonography machine.

## Methods

### Patients

Patients were identified in a prospective manner after multidisciplinary team (MDT) discussion. The surgeon in charge of their case (AR or RUA) highlighted to the anesthetist that intraoperative localization was required.

### Technique

**Figure 1:**
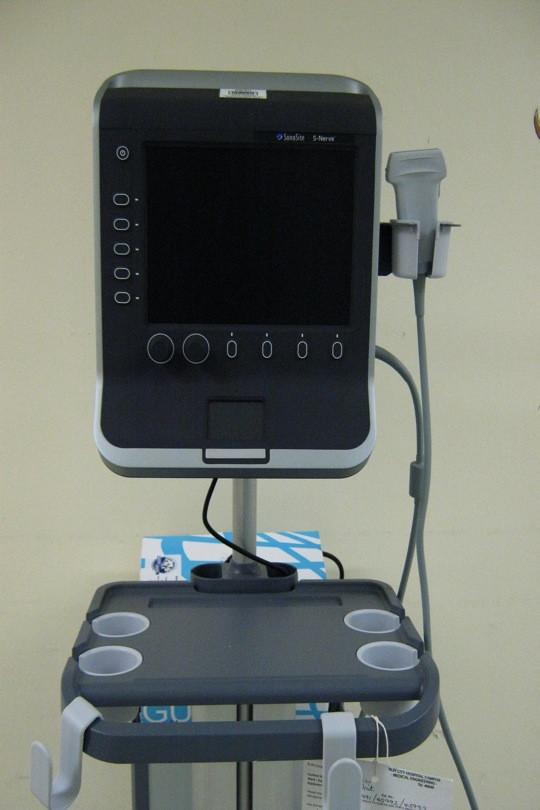
Sonosite S-Nerve Portable Ultrasound.

Our initial patient group consisted of seven patients (Table 1), on whom we carried out portable ultrasonography (Sonosite® S-Nerve; SonoSite Inc, Bothell, WA, USA) during their surgery. Once the patient was anesthetized, the suspected site of the tumor was scanned on the instrument’s nerve settings (Figure 1) with a 38 mm linear transducer (5 to 10 MHz). The tumor was identified by its sonographic appearance relative to the surrounding tissues. The incision site was then planned and the tumor identified surgically. We choose to use the portable ultrasonography examination before skin preparation, although sterile sheaths for this machine are available to facilitate intraoperative use.

**Table 1:** Demographics, tumor site, size, and pathology results for patients included in this study

**Patient number**	**Age/Sex **	**Site**	**Maximum tumor dimension on imaging, mm**	**Final histopathological diagnosis**
1	61/M	Forearm (BEA^1^ stump)	20	Neuroma
2	53/M	Forearm (BEA^1^ stump)	20	Intraneural perineuroma
3	18/M	Popliteal fossa	16	Glomus tumor
4	2/F	Thigh	28	Rhabdomyosarcoma
5	44/F	Forearm	10	Schwannoma
6	46/M	Popliteal fossa	16	Schwannoma
7	40/F	Shoulder girdle	20	Malignant peripheral nerve-sheath tumor

^**1**^Below-elbow amputation.

## Results

### Patient demographics

The demographics of our patient group is summarized in Table 1. We initially operated on seven patients (mean age 38 years, range 2 to 61 years). There was a slight male predominance. All the tumors were in a limb or limb girdle, with a mean tumor diameter of 18 mm (range 10 to 28 mm). Five cases were ultimately assessed as benign and two malignant. One of our patients had induction chemotherapy before the operation to reduce the size of the tumor.

### Patients

**Figure 2:**
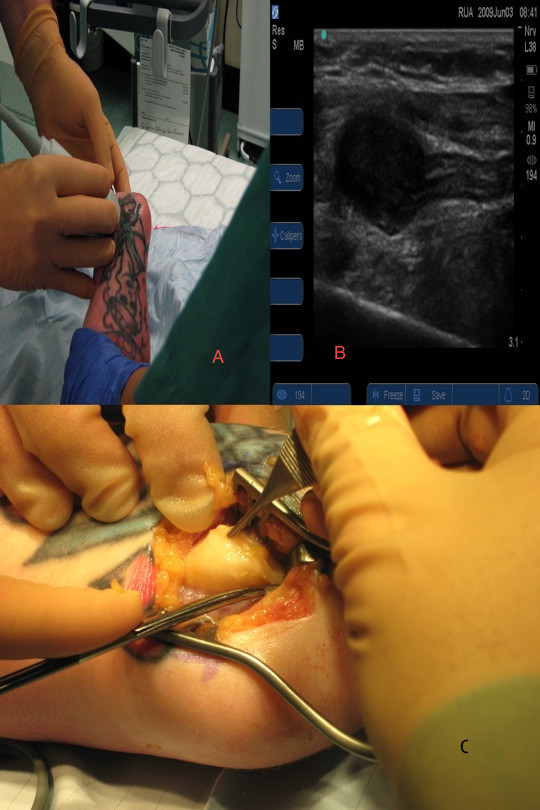
**Localization of tumor and surgical excision of neuroma.** [rvm1] (**A**) Ultrasonographic localization of tumour, (**B**) ultrasonographic images, (**C**) surgical excision of neuroma following ultrasonographic localisation.

Patients 1 and 2 had both been treated previously by below-elbow amputation for sarcomas. On surveillance MRI scans solid lesions were noted, raising the possibility of local recurrence. However, in each case no tumor was palpable on clinical examination. After MDT discussion, it was decided to perform marginal surgical excision of these tumors. Although the MRI scan gave an idea of the level of these tumors, it was difficult to plan their surgical approach. The tumors were therefore localized (Figure 2), the most appropriate incision site planned, and the tumors successfully excised.

Patients 3 and 6 presented with posterior knee pain and a small tumor was identified by MRI and ultrasonography. Patient 4 was a 18-month-old child with an embryonal rhabdomyosarcoma in her posterior thigh, which became impalpable after treatment with induction chemotherapy. Patient 5 was a woman with a deep nerve-sheath tumor of her forearm. Patient 7 had undergone an unplanned excision of a sarcoma, and MRI identified impalpable tumor residue. All surgical excisions were complete.

### Outcome

In all seven cases, the tumors were well-visualized intraoperatively by portable ultrasonography, and appropriate targeted surgery was performed. No patient required further surgery, and there were no complications of the technique. There have been no local recurrences, although the longest follow-up thus far is only 2 years.

No additional pre-operative investigations were required on the day for any of our patients. This method did not add any significant length to the operating time, as our experienced anesthetist (JAD) performed the procedure.

## Discussion

The mainstays of investigations for the evaluation of soft-tissue tumors are ultrasonography and MRI [1]. MRI plays a vital role in the investigation of these lesions because of its accuracy in localizing the tumors and also in assessing the extent of the tumors and their degree of invasion. MRI is thus the primary investigative method to evaluate soft-tissue sarcomas.

Most musculoskeletal tumors are palpable. Obtaining initial tissue diagnosis and planning their surgical excision is therefore not unduly challenging. In some cases, where a recurrence is suspected or the primary tumor is impalpable, a method of radiological evaluation on the day of the surgery is useful to plan the incision site and successful treatment.

Pre-operative fine-wire localization has traditionally been the most popular method in localization of impalpable tumors, especially in the case of breast lesions [9] and impalpable soft-tissue tumors [10], and also in localization of intrathoracic lung lesions [11]. However, this process involves inconvenience to the patient because of the pain and discomfort involved, and to the hospital team in terms of time consumption and organization [12]. This method is also associated with the risk of dislodgement of the wire during preparation and surgical positioning, which results in failed excision and potential tumor seeding along the needle track [12]. Thus, a non-invasive method of localization is preferred in the treatment of impalpable lesions.

High-resolution ultrasonography is a successful method of analyzing small tumors, including recurrences in soft tissue and subcutaneous planes [13]. Because of the portable nature of the ultrasonography machines, they can be used intraoperatively to identify and evaluate the suspected lesions.

Fornage *et al*. [12] studied this method for intraoperative localization of breast lumps in 26 patients. In addition, they also used this technique to confirm these lumps by scanning the specimens after removal. They reported successful localization and confirmation in all of their patients. Confirmation of the excision by scanning was useful because two of the specimens were not found during the first excision, and they proceeded to further excision and confirmation in the same setting. They concluded that this method is effective and successful, and reduces inconvenience to the surgical team and the patient.

In addition, high-resolution ultrasonography has been successful in assisting with accurate localization and collection of tissue specimens to aid with diagnosis [13]. We have also used portable ultrasonography regularly to target core needle biopsies of sarcomas, with similar success, eliminating the need in some cases for radiological referral to obtain tissue diagnosis and subsequent treatment for the patient.

We believe that portable ultrasonography machines are a valuable adjunct to perioperative localization of impalpable soft-tissue tumors, and this use could be transferred to enable accurate targeting of soft-tissue tumors in the outpatient department. This can alleviate the need for radiologist presence in obtaining tissue diagnosis and at the time of surgery. We have been unable to find any other studies of portable ultrasonography use in the intraoperative evaluation of soft-tissue tumors as an aid to planning surgical approach, despite an extensive literature search using PubMed and MEDLINE.

Studies have been carried out to evaluate the efficacy of portable ultrasonography in detecting soft-tissue foreign bodies, which have rated the overall sensitivity as 89% and specificity as 93%s [14]. In spite of the high sensitivity and specificity rates documented, there is evidence that in the hands of inexperienced operators, the use of portable ultrasonography is neither sensitive nor specific [14].

In our study group, we had an anesthetist with expertise in using the portable ultrasonography to guide us through the tumor localization process. Although use of ultrasonography is associated with a learning curve, we believe that with appropriate training, guidance from an experienced anesthetist available at the time of surgery, and regular use, portable ultrasonography machines can be a valuable adjunct in the intraoperative localization of impalpable tumors and also in obtaining tissue samples for diagnosis in the outpatient department.

## Conclusion

Intraoperative ultrasonography examination using a portable machine is a simple and practical alternative to a formal ultrasonography scan for localization of soft tissue tumors and can prevent delay in patient treatment.

### Consent

In accordance with United Kingdom National Research Ethics Service advice, formal ethical approval was not sought because the technique was both non-invasive and a review of standard clinical practice. All patient data is anonymised and no patient identifiable images are used.

## Competing interests

The authors declare that they have no competing interests.

## Authors’ contributions

JJ wrote the first draft, RUA conceived the idea, JAD performed the scanning, and AR and RUA carried out the surgeries and helped collect clinical materials. All authors read and approved the final manuscript.
